# Prediction of nuclear proteins using SVM and HMM models

**DOI:** 10.1186/1471-2105-10-22

**Published:** 2009-01-19

**Authors:** Manish Kumar, Gajendra PS Raghava

**Affiliations:** 1 Bioinformatics Centre, Institute of Microbial Technology, Sector 39A, Chandigarh-160036, India

## Abstract

**Background:**

The nucleus, a highly organized organelle, plays important role in cellular homeostasis. The nuclear proteins are crucial for chromosomal maintenance/segregation, gene expression, RNA processing/export, and many other processes. Several methods have been developed for predicting the nuclear proteins in the past. The aim of the present study is to develop a new method for predicting nuclear proteins with higher accuracy.

**Results:**

All modules were trained and tested on a non-redundant dataset and evaluated using five-fold cross-validation technique. Firstly, Support Vector Machines (SVM) based modules have been developed using amino acid and dipeptide compositions and achieved a Mathews correlation coefficient (MCC) of 0.59 and 0.61 respectively. Secondly, we have developed SVM modules using split amino acid compositions (SAAC) and achieved the maximum MCC of 0.66. Thirdly, a hidden Markov model (HMM) based module/profile was developed for searching exclusively nuclear and non-nuclear domains in a protein. Finally, a hybrid module was developed by combining SVM module and HMM profile and achieved a MCC of 0.87 with an accuracy of 94.61%. This method performs better than the existing methods when evaluated on blind/independent datasets. Our method estimated 31.51%, 21.89%, 26.31%, 25.72% and 24.95% of the proteins as nuclear proteins in *Saccharomyces cerevisiae, Caenorhabditis elegans, Drosophila melanogaster*, mouse and human proteomes respectively. Based on the above modules, we have developed a web server NpPred for predicting nuclear proteins .

**Conclusion:**

This study describes a highly accurate method for predicting nuclear proteins. SVM module has been developed for the first time using SAAC for predicting nuclear proteins, where amino acid composition of N-terminus and the remaining protein were computed separately. In addition, our study is a first documentation where exclusively nuclear and non-nuclear domains have been identified and used for predicting nuclear proteins. The performance of the method improved further by combining both approaches together.

## Background

The genome of the large number of organisms has been completely sequenced or in the final stage of completion due to the advancement in the technology. Thus, the functional annotation of proteomes is one of the major challenges in the post genomic era as the numbers of protein with known sequences are growing at exponential rate. The experimental techniques for assigning the functions are slow, costly and cumbersome. In order to assist the biologists in functional annotation of the proteomes, large number of computational methods has been developed. Similarity search is one of the most commonly-used techniques for assigning the function of a newly sequenced protein. However, it fails if query/new protein does not have sequence similarity with a protein whose function is known.

One of the indirect techniques that are used for assigning function of a protein is the prediction of its subcellular localization. As the function of a protein is closely related to its cellular attributes, the related proteins must be localized in the same cellular compartment to cooperate toward a common function. In the past, large number methods have been developed for predicting the subcellular localization of proteins and most of them were developed for predicting multiple locations. Though multi-location prediction methods provide comprehensive information, they are not optimized for a particular location. Hence, recent studies are focused on the development of methods for predicting proteins in specific location [1-3].

One of the important compartments of a eukaryotic cell is nucleus, which is essential for regulating various biological activities. Thus there is a need to develop an accurate method for predicting nuclear proteins. In the past, several methods have been developed for predicting nuclear proteins. PredictNLS was the first method developed using Nuclear Localization Signal (NLS) [4]. Heddad et al developed a genetic programming based method NucPred that tried to compile a list of potential NLSs [5]. Recently NucPred has been evaluated on a new dataset [6] and its performance was found to be better than the PredictNLS, LOCtree [7] and BaCelLo [8] (generalized subcellular localization methods). In the present work, a systematic attempt has been made to predict nuclear proteins with high accuracy. All the nuclear and non-nuclear proteins have been analyzed in order to understand major features of the nuclear proteins. Based on these observations, SVM based modules have been developed for predicting nuclear proteins. In addition, we have developed HMM based module which used both exclusive nuclear and non-nuclear domains.

## Results

### Analysis of amino acid composition

**Figure 1:**
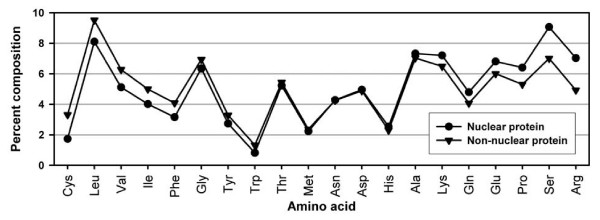
**Average amino acid composition of nuclear and non-nuclear protein sequences of main dataset**.

As shown in Figure 1, the composition of few amino acids differs significantly in the nuclear and non-nuclear proteins. In the nuclear proteins, aromatic (Tyr, Phe) and non-polar aliphatic (Leu, Val, Ile) residues are less abundant. Charged residues Lys, Glu, Arg are more prevalent in the nuclear proteins compared with the non-nuclear proteins. Among the polar residues, Gln and Ser were abundant in nuclear proteins and Cys in non-nuclear proteins. We have also analyzed the statistical significance of the differences in amino acid compositions of the amino acid whose difference is more than 0.5 (amino acids other than Thr, Met, Asn, Asp, His, Ala) at confidence level 0.001. We found that differences between14 types of amino acid are statistically significant at the confidence level 0.001.

### SVM modules of amino acid and dipeptide composition

The above analysis indicates that the nuclear and non-nuclear proteins can be discriminated based on their composition. Hence we have developed a SVM-based module using amino acid composition for predicting nuclear protein and achieved the maximum MCC of 0.59 with polynomial kernel (Table 1). In our dataset, the ratio of nuclear and non-nuclear proteins was approximately 1:4. Therefore, it was possible to achieve an accuracy of about 80%, simply by predicting all protein of testing set as non-nuclear proteins. In order to avoid this sort of random prediction, we considered MCC as more meaningful measure than simple accuracy because it also considers the under-prediction of nuclear and non-nuclear proteins [9]. We have considered optimal performance at the threshold where sensitivity and specificity were nearly equal.

**Table 1:** The performance of SVM models using various types of composition.

**Composition Type**	**Sensitivity**	**Specificity**	**Accuracy**	**MCC**
**Amino Acids**	81.33	81.75	81.64	0.59
**Dipeptides**	82.03	83.11	82.83	0.61
**SAAC 2-parts (equal)**	82.18	82.68	82.55	0.61
**NT15+R**	83.03	85.76	85.05	0.65
**NT25+R**	**85.50**	**85.46**	**85.47**	**0.66**
**NT35+R**	83.69	83.89	83.84	0.63
**CT15+R**	77.41	82.06	80.84	0.56
**CT25+R**	80.29	80.93	80.77	0.57
**CT35+R**	80.19	81.22	80.95	0.57
**SAAC 3-parts (equal)**	81.48	85.04	84.11	0.63
**NT15+R+CT15**	83.62	83.65	83.64	0.63
**NT25+R+CT25**	***83.69***	***85.91***	***85.33***	***0.66***
**NT35+R+CT35**	83.77	84.03	83.96	0.63
**NT45 +R+CT45**	82.77	83.95	83.64	0.62
**SAAC 4-parts (equal)**	83.80	83.47	83.55	0.63

In split amino acid composition whole protein was divided into X {X = 2,3,4} equal parts; amino acid composition of each fragments was determined individually and concatenated together to make final input vector of dimension 20*X. NT15 = amino acid composition of N-terminal 15 residues, CT15 = amino acid composition of C-terminal 15 residues and so on; R = amino acid composition of remaining residues.

Previouly, the dipeptide composition has been successfully used for predicting subcellular localization of proteins [10,11]. Hence in this study, we have developed SVM module using dipeptide composition and achieved the maximum MCC of 0.61 (with overall accuracy 82.83%) using polynomial kernel. This showed that the performance of SVM model based on dipeptide was better than that based on amino acid composition.

### Split amino acid composition (SAAC)

**Figure 2:**
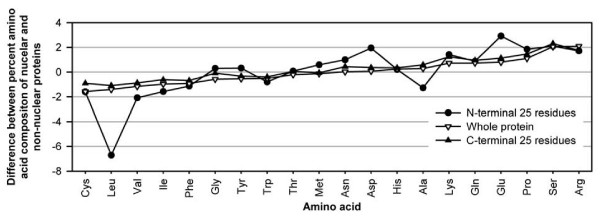
**Variation in the preference of amino acids at N-terminal 25 residues, C-terminal 25 residues and full length nuclear and non-nuclear proteins**.

Previously, it has been shown that amino acid compositions of non-overlapping fragment perform better than that of the whole protein due to increase in the information content [2,12,13]. In order to understand, the compositional biasness in the nuclear proteins we computed the difference in composition for all 20 amino acids in the nuclear and the non-nuclear proteins for entire protein, first 25 residues (N-terminus region) and last 25 residues (C-terminus region). As shown in Figure 2, compositional biasness is more prominent in the N-terminus region than the full-length protein or the C-terminus region. At the N-terminal region of the nuclear proteins, low frequency of Leu and Ala and high frequency of Asp and Glu were observed. Hence we used SAAC where the protein sequence was divided into parts and then composition of each part was computed separately. The SVM module takes the composition of each part as input vector. First, we developed a SVM model using the composition of two equal parts of a protein and achieved the MCC of 0.61. Secondly, SVM models have been developed using two unequal parts of a protein. Two strategies were adopted for dividing the sequences into unequal length a) first fragment contain few residues of N-terminal and second fragment contain remaining part and b) first fragment has few C-terminal residues and remaining residues in second fragment. In general, the performance of SVM models developed using N-terminal and the remaining residues was better than the other one (Table 1). It indicates that the N-terminal region of the nuclear proteins has some biasness in amino acid composition, which is not present at the C-terminal. This also supports our observation (Figure. 2), where compositional biasness of N-terminus residues is more prominent. Therefore SVM was able to discriminate among them more efficiently. We have developed several SAAC composition based modules and achieved the maximum MCC of 0.66 using the composition of first 25 residues and remaining ones (NT25+R). We have also developed 3-part SAAC based SVM models using equal and unequal length fragments. SVM model developed using the N-terminal 25, C-terminal 25 and remaining residues (NT25+R+CT25) showed the maximum MCC of 0.66. When the protein sequences were splitted into four parts of equal length, with SAAC we found a MCC of 0.63 which was similar to that obtained after dividing the proteins into two or three parts of equal length. As shown in Table 1, SVM models developed on NT25+R and NT25+R+CT25 have similar MCC. In subsequent studies, we have used only NT25+R based SVM models.

### Occurrence of Pfam domains

In this study we have developed a HMM based module for predicting nuclear proteins. We analyzed the nuclear and non-nuclear proteins in order to understand the occurrences of Pfam domains. All the sequences in "data_main" were searched against Pfam database using HMMER. We found, altogether, 2073 types of domains in our dataset of which 558 were found exclusively in the nuclear proteins (henceforth called as exclusive nuclear domains), 1197 only in the non-nuclear proteins (henceforth called as exclusive non-nuclear domains) and 159 in both type of proteins (henceforth called as shared domains). We built a domain database NucPfam containing all three types of domains [see Table S1, S2 and S3 and Figure S1 in Additional file 1]. In order to predict whether a protein can be localized in the nucleus or not, we performed HMMER search against NucPfam database. A protein was assigned nuclear if it has exclusive nuclear domain or non-nuclear protein if it has exclusive non-nuclear domain. Using this approach, we were able to predict 1858 nuclear and 6090 non-nuclear proteins. It was observed that there was no hit for large number of proteins (1305) due to the absence of exclusive domains and 1119 proteins contain only shared domains. Thus we developed a hybrid method, which combined SVM modules trained on NT25+R amino acid composition and occurrence of Pfam domain. In this hybrid method, a protein was predicted to be nuclear or non-nuclear on the basis of the presence of exclusive nuclear or non-nuclear domains. In the cases, where a protein did not have any exclusive domain, SVM module was used for prediction. The performance of this hybrid method was evaluated at different thresholds of SVM and achieved an accuracy of 94.61% with MCC of 0.87 (Table 2). The hybrid method is henceforth referred as NpPred.

**Table 2:** The performance of hybrid module, which combines HMM and SVM model (using NT25+R).

**SVM Threshold**	**Sensitivity**	**Specificity**	**Accuracy**	**MCC**
**-1.0**	99.19	88.03	90.95	0.80
**-0.9**	99.04	88.86	91.53	0.82
**-0.8**	98.86	89.61	92.03	0.82
**-0.7**	98.63	90.37	92.53	0.83
**-0.6**	98.34	91.08	92.98	0.84
**-0.5**	97.82	91.67	93.28	0.85
**-0.4**	97.49	92.39	93.72	0.85
**-0.3**	96.86	92.96	93.98	0.86
**-0.2**	96.13	93.41	94.12	0.86
**-0.1**	95.46	94.05	94.42	0.86
**0.0**	**94.80**	**94.55**	**94.61**	**0.87**
**0.1**	94.02	95.00	94.75	0.87
**0.2**	92.80	95.22	94.59	0.86
**0.3**	92.07	95.77	94.80	0.87
**0.4**	90.70	96.18	94.75	0.87
**0.5**	89.52	96.62	94.77	0.87
**0.6**	87.90	96.91	94.55	0.86
**0.7**	86.27	97.25	94.38	0.85
**0.8**	85.21	97.57	94.34	0.85
**0.9**	83.58	97.70	94.01	0.84
**1.0**	82.66	98.00	93.99	0.84

Threshold is for cut-off for SVM on the basis of which performance is calculated. Values in bold shows the region where sensitivity and specificity are approximately equal.

### Benchmarking of methods

It is important to compare the performance of a newly developed method with the existing methods. In the past, a large number of methods have been developed for predicting the subcellular localization of the proteins and those could be used for predicting the nuclear proteins. It is not practically possible to compare the performance with all theese methods. Recently, Pierleoni et al. [8] evaluated the performance of many 'subcellular localization' methods. Thus, we have evaluated the performance of NpPred on the same dataset (Blind1 and Blind2). As shown in the Table 3, NpPred performed better than other methods on both animal (Blind1 dataset) and fungal proteins (Blind2 dataset). These results demonstrate that the models developed in this study are better than the existing methods. All these methods were developed for multiple locations and hence they were not optimized for nuclear proteins. Recently, Brameier et al [6] have developed a method 'NucPred' for predicting nuclear protein and compared the performance of their method with other existing methods. Here, we evaluated the performance of our models on the same dataset (Blind3). As shown in the Table 4, NpPred achieved 0.83 (83%) sensitivity, which is better than the performance of the existing methods.

**Table 3:** The performance of different subcellular localization methods on blind/independent dataset used in BaCelLo.

	***Blind1 Dataset *(Animal Proteins)**			***Blind2 Dataset *(Fungal Proteins)**		
**Method**	Cov*	***nAcc***	***GAv***	***Cov***	***nAcc***	***GAv***

**BaCelLo**	66.1	56.4	61.1	66.4	71.3	68.8
**Loctree**	62.2	49.5	55.5	66.4	66.9	66.6
**Psort II**	70.2	43.0	54.9	71.1	44.2	56.1
**SubLoc**	67.8	37.2	50.2	70.5	38.4	52.0
**ESLpred**	79.1	35.8	53.2	84.4	37.5	56.3
**LOCSVMpsi**	80.2	38.7	55.7	88.5	51.0	67.2
**pTARGET**	73.3	64.2	68.6	62.3	63.5	62.9
**NpPred**	**87.3**	**74.3**	**80.5**	**93.4**	**72.7**	**82.4**

Cov is coverage/sensitivity; nAcc is normalized accuracy; GAv = geometric average between coverage and normalized accuracy (*see Additional file 1 for detail description of these parameters).

**Table 4:** The performance of nuclear protein prediction methods on 2213 human proteins (1526 non-nuclear and 687 nuclear).

**Method**	**Sensitivity**	**PPV** **(Probability of correct** **prediction of nuclear proteins)**
**NucPred (0.8 threshold)**	0.31	0.62
**NucPred (0.5 threshold)**	0.63	0.48
**PredictNLS**	0.23	0.63
**PSORT II**	0.70	0.47
**NucPred (0.8) AND PredictNLS**	0.17	0.73
**NucPred (0.8) OR PredictNLS**	0.43	0.57
**LOCtree**	0.63	0.59
**BaCelLo**	0.61	0.67
**NpPred**	**0.83**	**0.63**

PPV = Tp/Tp+Fp; Tp = True positive predictions; Fp = False positive predictions.

### Webserver

A webserver NpPred has been developed for predicting nuclear proteins. It allows users to submit up to 1000 sequences at a time for prediction. NpPred has been developed using programming language Perl, CGI-perl and HTML, launch on SUN server T1000 under Solaris 10.0 environment. This server is available from URL  for academic users [see Figure S2 in Additional file 1].

The prediction results are displayed in a user-friendly format. The result page first displays the prediction parameters like approach of prediction, SVM threshold, e-value of Pfam domain search. In case of only SVM based prediction, SVM score along with prediction result is displayed [see Figure S3 in Additional file 1]. The result page of SVM and Pfam based hybrid approach prediction, Pfam domain and their nature of existence (exclusive nuclear/non-nuclear), SVM score and final prediction of each query sequence will be displayed [see Figure S4 in Additional file 1].

### Proteome annotation

**Figure 3:**
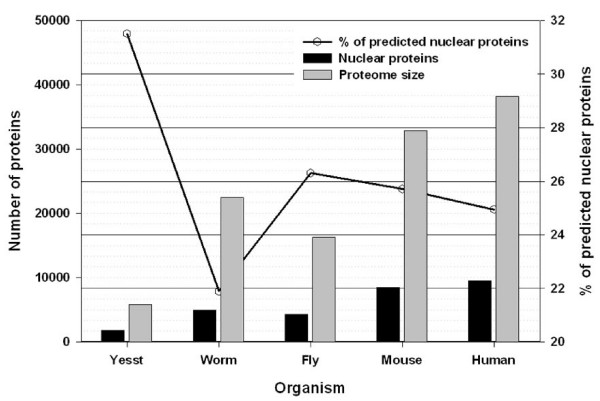
**Prediction of nuclear proteins using NpPred in proteome of Yeast (*S. cerevisiae*), Worm (*C. elegans*), Fly (*D. melanogaster*), Mouse (*M. musculus*) and Human (*H. sapiens*)**.

We predicted the number of nuclear proteins in five complete proteomes. The list includes single cell eukaryote (*S. cerevisiae*), invertebrates (*C. elegans, D. melanogaster*) to highly evolved mouse and human. In order to avoid the large number of false positives during proteome wide prediction, we used a slightly higher SVM prediction cut-off (1.0) to classify a protein into nuclear protein by SVM. But e-value threshold for HMM based Pfam search remain the same as 1e^-5^. In *S. cerevisiae *surprisingly we found the highest fraction of nuclear proteins (31.51% of total proteome; 1821 proteins in total). Among other four organisms the fraction of nuclear proteins lies between 22–26% of total proteome size. In *C. elegans *and *D. melanogaster *NpPred predicted 21.89 (4911 proteins) and 26.31% (4275 proteins) of total proteins as nuclear proteins, respectively. On the other hand 9533 and 8460 (24.95 and 25.72% of total proteome) proteins were predicted as nuclear resident protein in human and mouse respectively. The number of predicted nuclear proteins and proteome size has been shown in Figure 3. Proteome-wide list of NpPred predicted nuclear proteins could be found at NpPred server .

## Discussion

Within each subcellular compartment of a given cell type, proteins have co-evolved according to the surrounding physico-chemical environment. However, the general features of the nuclear environment have been constant factors throughout eukaryotic evolution. These factors pose environmental constraints on the evolution of protein sequence and structure so that the proteins will have to adapt to the different environmental constraints. If this hypothesis is true then instead of simply searching the single amino acid sequence, the better approach is to search stretch of sequences that are known to be conserved in similar types of proteins. Taken this into consideration, we have used Pfam domain database and extracted three type of domains namely exclusive nuclear, exclusive non-nuclear and shared. Proteins having exclusive nuclear or non-nuclear domain, were predicted as nuclear and non-nuclear proteins respectively. This approach was able to predict only 1858 proteins as nuclear. In first impression, it seems that the profile search approach is not very efficient. But if we also consider the proteins which were not classified into any class due to the presence of shared domains then it is clear that this approach has the capability to filter out all sequence which can be wrongly classified by BLAST. Because if BLAST search was done on proteins having shared domain they might be classified wrongly as non-nuclear. In reality, these proteins may not either present in nucleus or having only a transient stay. Hence the actual challenge is to increase the coverage and decrease the number of false predictions.

Among all the cellular organelles of eukaryotic cell, nucleus is very interesting organelle. Unlike other organelles it is not strictly isolated from cytoplasm due to the presence of nuclear pore complexes. Nuclear pore complex is permeable to small (<5 kDa) neutral molecules [14]. Presence of chromosome and their regulatory proteins make nucleus the central point of gene regulation. Hence prediction of nuclear proteins can be an important step in understanding of their function and building protein networks. In this study, an attempt has been made to improve the accuracy of nuclear protein prediction. First we analyzed and compared the composition of nuclear and non-nuclear proteins. It was observed that certain amino acids are more prominent in nuclear proteins where as few others are more prominent in non-nuclear proteins. This observation reveals the possibility of discriminating nuclear and non-nuclear proteins on the basis of amino acid composition. Based on these observations, SVM models have been developed using amino acid composition and achieved a reasonable accuracy. It has been shown in the past that dipeptide composition based models perform better than amino acid composition based models because dipeptide also provides information about local order. As shown in Table 1, SVM model based on dipeptide was better than amino acid composition based model. Due to the presence of NLS, the first logical step in prediction of nuclear protein is to search for signal peptides in the sequence. But it has been shown in previous studies that prediction coverage by using NLS is very low [4]. Moreover we can miss the proteins, which do not have NLS or are transported into nucleus as a complex with other protein. The alternative approach would be to infer location by the sequence homology to a protein with known location. Similarity based annotation is said to be highly accurate if an experimentally annotated homologous protein is present in the database. But in their study, Cokol et al [4] have observed different scenario. They have found about 30 protein pairs with >80% sequence identity and different subcellular location. This shows that there is a possibility of interpreting wrongly even if searching is done on a very clean and experimentally annotated data. The chances of wrong annotation by BLAST search increases many folds if a general database such as SWISS-PROT is used. In addition there is also a chance of not getting any hit during BLAST search which results in the reduction of total proteome size. This shows the limitation of BLAST searching.

In order to increase the coverage we have developed SVM modules based on different form of amino acid composition. We have found maximum performance with NT25+R composition based model. In order to exploit the benefits of generalized SVM based prediction as well as profile search, we have also developed hybrid prediction modules NpPred.

NpPred was also evaluated in comparison with different 'subcellular localization' prediction methods using two independent datasets. First dataset contains fungi and animal proteins extracted from SWISS-PROT release 41 to 48 [8]. Our training dataset (data_main) contain protein sequences only up to SWISS-PROT release 40.41. It means that the independent dataset sequences were not included during five-fold cross-validation phase. On this dataset performance of NpPred is superior to seven other prediction methods. The second independent dataset contains only human protein sequences, which were earlier used for benchmarking of NucPred [6]. Even on this dataset NpPred performed better than the other five methods. All these demonstrate that the method described in this study, performs better than the existing subcellular localization methods for the prediction of the nuclear proteins. In summary, this method will complement the existing 'subcellular localization' methods in prediction of nuclear proteins.

## Conclusion

The nucleus is a highly complex organelle that houses the genome and their corresponding regulatory factors. Hence the prediction of nuclear proteins can be an important step towards understanding the gene regulatory mechanism and their interactions. We developed a highly accurate genome-scale nuclear proteins prediction method NpPred. First a domain database NucPfam has been developed that classifies the domains on the basis of their occurrence in nuclear and non-nuclear proteins. In case a protein contains no domains then SVM module was used for prediction. The five-fold cross-validation method showed an accuracy of 94.61% using NpPred. Furthermore, NpPred was used to predict the nuclear proteins in five representative proteomes. These genome-scale predictions and NucPfam domain database can provide an excellent starting point for experimentalists to improve the functional annotation of proteins. A web-server NpPred has also been developed to make the prediction method available to the scientific community. We hope that NpPred would able to expedite the rate of protein function prediction. The only limitation we could perceive in the present work is that we have considered only the steady-state localizations of the proteins and did not take in to account the proteins that enter the nucleus in a transient or temporally regulated manner. Therefore, the algorithm developed is only aimed at finding proteins that are nuclear at steady-state. An ideal method should address the transient localization also.

## Methods

### Datasets

#### Main dataset (data_main)

The selection of dataset is the most important consideration during development of a prediction method. The sequence used for training should have high quality curation and should not contain the proteins belonging to gene families and homologous genes from various organisms. If the proteins in the dataset have high similarity among each other then the method will show very high accuracy during training but it will be not very effective during real life prediction. Hence the final dataset is created in such a way that representative proteins will not have sequence similarity more than a certain threshold limit. This type of dataset is called as non-redundant dataset. In this work we used the non-redundant database of 10372 eukaryotic proteins obtained from Guda et al, 2004 [1]. This dataset has been used earlier for developing MITOPRED [1] and Mitpred [2]. Originally these sequences were extracted from Swiss-Prot release 40.41 . It consists of proteins having experimentally determined subcellular locations (cytoplasm = 1712, nucleus = 2710, mitochondria = 1432, extracellular or secretory = 3471, endoplasmic reticulum = 644, plasma membrane = 108, golgi complex = 142 and peroxisome = 153). Sequences with low quality annotation such as 'by similarity', 'potential', 'probable' and 'possible' were not included in the dataset. In this study, 2710 nuclear proteins were used as positive example and remaining 7662 as negative examples.

#### Blind or independent dataset

We use three different types of blind datasets obtained from different sources i) Blind1 dataset have 363 nuclear and 344 non-nuclear animal proteins, earlier used in BaCelLo for benchmarking of different eukaryotic subcellular localization methods [8], ii) Blind2 dataset have 122 nuclear and 57 non-nuclear fungal proteins also used in BaCelLo [8] and iii) Blind3 dataset consists of 687 nuclear and 1526 non-nuclear human proteins used for benchmarking NucPred [6].

### Performance evaluation and parameters

Jackknife or leave one out cross-validation is considered to be the most rigorous test for evaluation of performance [15]. But it usually takes very long time to perform jackknife test. As a compromise, we use the less rigorous 5-fold cross-validation where proteins of each class were randomly divided into five sets [2,11,16]. Four parts were used for training and remaining one part for testing. This process was repeated five times so that each set was used once for testing.

For performance evaluation we used standard parameters routinely used in other prediction methods [2,11,16]. Followings are the brief description of these parameters; i) sensitivity is percentage of correctly predicted nuclear proteins; ii) specificity is percentage of correctly predicted non-nuclear proteins and iii) accuracy is percent of correctly predicted nuclear and non-nuclear proteins in whole data and iv) Matthews correlation coefficient (*MCC*) is the statistical parameter to access the quality of prediction and taking care of unbalancing in data [17]. *MCC *equal to 1 is regarded as perfect prediction, 0 for completely random prediction and -1 as the worst possible prediction. These parameters can be calculated using following equations

(1) $$Sensitivity=\frac{TP}{TP+FN}\times 100$$

(2) $$Specificity=\frac{TN}{TN+FP}\times 100$$

(3) $$Accuracy=\frac{TP+TN}{TP+TN+FP+FN}\times 100$$

(4) $$MCC=\frac{\left(TP\times TN)-(FP\times FN\right)}{\sqrt{\left(TP+FP)(TP+FN)(TN+FP)(TN+FN\right)}}$$

Where *TP *and *TN *are correctly predicted positive (nuclear) and negative (non-nuclear) proteins respectively. *FP *and *FN *are wrongly predicted nuclear and non-nuclear proteins respectively.

### Residue composition

Amino acid composition is the fraction of each amino acid type within a protein. In case of amino acid composition a vector of dimension 20 represents a protein. In order to include local order information, we also compute dipeptide composition of proteins, where dimension of vector 400 represents a protein. Following equations were used to compute amino acid and dipeptide compositions.

(5) $$comp(i)=\frac{R_{i}}{N}\times 100$$

(6) $$dpep(j)=\frac{D_{j}}{N-1}\times 100$$

Where *comp(i) *and *dpep(j) *are amino acid and dipeptide composition of residue type *i *and dipeptide of type *j *. *N *is total number of amino acids in protein.

### Split amino acid composition

In the case of split amino acid composition, protein sequence was divided into non-overlapping fragments then composition of each fragment was calculated independently. Thus the dimension of final input vector will be n × 20 dimensions, where n is number of fragments. In this study, proteins were divided into (i) two parts (ii) three parts and (iv) four parts.

### Composition of terminal residues

It has been observed that protein may have localization signal at N- or C-terminus. In order to exploit this knowledge, we developed models using N-terminus or C-terminus composition and composition of remaining portion of a protein.

### Support Vector Machine

In this study we implemented SVM using SVM_light package , which allows us to choose a number of parameters and kernels (e.g. linear, polynomial, radial basis function, sigmoid or any user-defined kernel). The selection of kernel is very important in SVM, which is analogous to choose architecture in ANN. In this study we used linear, polynomial and RBF kernels. For detail descriptions of SVM please refer [18].

### Occurrence of Pfam domains

In the present study, hidden Markov model (HMM) based searching was implemented using HMMER  and proteins were searched against Pfam domain database [19] (version 21.0). Pfam contains multiple sequence alignments and hidden Markov models of protein domains and families. Each protein of data_main was searched against Pfam database using HMMER at e-value of 1e-5. Search results were analyzed to detect three type of domains; (i) exclusive nuclear domains (occurs only in nuclear proteins) (ii) exclusive non-nuclear domains (found only in non-nuclear proteins) and (iii) shared domains (present in both type of proteins). A protein was assigned nuclear or non-nuclear protein if it contains exclusive nuclear or non-nuclear domain respectively.

### Annotation of proteomes

We annotate five eukaryotic proteomes using the method developed in this study. These proteomes *S. cerevisae, C. elegans, D. melanogaster, M. musculus *and *H. sapiens *were downloaded from EBI  and contains 5780, 22437, 16251, 32895 and 38213 proteins respectively.

## Authors' contributions

MKR carried out the data analysis and interpretation, developed computer programs, wrote the manuscript and developed the web server. GPSR conceived and coordinated the project, guided its conception and design, helped in interpretation of data, refined the drafted manuscript and gave overall supervision to the project. Both authors read and approved the final manuscript.

## Availability and requirements

The NpPred prediction system using SVM and HMM based Pfam search for nuclear proteins prediction has been implemented at . In order to use NpPred the user should have access to the internet. Using our prediction server the user can do prediction of nuclear proteins.

## Supplementary Material

Additional File 1 **Additional results of nuclear protein prediction method.** The data provided description of various parameters used to evaluate blind1 and blind2 datasets, list of exclusive nuclear, non-nuclear and shared domains, their distribution statistics, and description of NpPred web-server. Click here for file
