# Supplementary material for: Prediction of nuclear proteins using SVM and HMM models
Source: BMC Bioinformatics. 2009 Jan 19;10:22. doi: 10.1186/1471-2105-10-22 (PMC2632991; doi:10.1186/1471-2105-10-22)
Supplement: Additional File 1 — Additional results of nuclear protein prediction method. The data provided description of various parameters used to evaluate blind1 and blind2 datasets, list of exclusive nuclear, non-nuclear and shared domains, their distribution statistics, and description of NpPred web-server. [file 1471-2105-10-22-S1.pdf]

Supplementary material

Prediction of Nuclear Proteins using SVM and HMM Models

Manish Kumar[1], Gajendra PS Raghava[1§]

[1]Bioinformatics Centre, Institute of Microbial Technology, Sector 39A, Chandigarh-160036, India. E-mail: raghava@imtech.res.in. Tel. no. 91-172-2690557; Fax 91-172-2690585
[§]Corresponding Author

Calculation of accuracy and normalized accuracy

Let us suppose that there are two classes of proteins, one class has 10 examples (Positive) and one with 1000 (Negative). Let further suppose that the prediction comes as

True Positive	False Negative	False Positive	True Negative
6	4	10	990

The standard accuracy for the positive class would be:
Acc= (6)/(6+10)= 37.5%

The normalized accuracy will be
nAcc= (6/10)/(6/10 + 10/1000) = 98.4%

For detail description please see:

Pierleoni A, Martelli PL, Fariselli P, Casadio R: **BaCelLo: a balanced subcellular localization predictor**. *Bioinformatics* 2006, **22**(14):e408-416.

Table S1: List of shared Pfam domains.

1.	AAA	43.	FH2
2.	AAA_5	44.	FKBP_C
3.	Acetyltransf_1	45.	Filament
4.	Actin	46.	GED
5.	Adap_comp_sub	47.	GTP_EFTU
6.	Amidohydro_1	48.	GTP_EFTU_D2
7.	Ank	49.	GTP_EFTU_D3
8.	Arf	50.	Gal-bind_lectin
9.	ArfGap	51.	HATPase_c
10.	Arm	52.	HEAT
11.	B56	53.	HECT
12.	BACK	54.	HIN
13.	BH4	55.	HMG_box
14.	BIR	56.	HSF_DNA-bind
15.	BTB	57.	Helicase_C
16.	Bcl-2	58.	HhH-GPD
17.	Bystin	59.	Hydrolase
18.	C1_1	60.	I-set
19.	C2	61.	IBN_N
20.	CARD	62.	IFP_35_N
21.	CBS	63.	IF_tail
22.	CRAL_TRIO	64.	KH_1
23.	CRAL_TRIO_N	65.	Kelch_1
24.	CSD	66.	Kelch_2
25.	CUB	67.	Kinesin
26.	Collagen	68.	Kunitz_BPTI
27.	DAGK_acc	69.	LBP_BPI_CETP
28.	DAGK_cat	70.	LBP_BPI_CETP_C
29.	DEAD	71.	LIM
30.	DNA_gyraseB	72.	LRR_1
31.	DNA_topoisoIV	73.	Lactamase_B
32.	DSPc	74.	LisH
33.	DUF1055	75.	M
34.	DnaJ	76.	MATH
35.	DnaJ_C	77.	MEF2_binding
36.	DnaJ_CXXCXGXG	78.	MH1
37.	Dynamin_M	79.	MH2
38.	Dynamin_N	80.	MIF4G
39.	EFG_C	81.	MMR_HSR1
40.	EFG_IV	82.	Mad3_BUB1_I
41.	ERG4_ERG24	83.	Metallophos
42.	Exo_endo_phos	84.	Miro
85.	F-box	123.	NHL

86.	NID	124.	SAM_1
87.	NTF2	125.	SAM_2
88.	NumbF	126.	SAND
89.	PA	127.	SH2
90.	PAAD_DAPIN	128.	SH3_1
91.	PABP	129.	SH3_2
92.	PAS	130.	Sec63
93.	PAS_3	131.	Synuclein
94.	PB1	132.	TACC
95.	PDZ	133.	TIG
96.	PH	134.	TPR_1
97.	PID	135.	TPR_2
98.	POLO_box	136.	TPR_MLP1_2
99.	PP2C	137.	Tap-RNA_bind
100.	PUA	138.	Tubulin
101.	PWWP	139.	Tubulin_C
102.	PX	140.	UBA
103.	Peptidase_C48	141.	UCH
104.	Peptidase_M1	142.	UQ_con
105.	Pkinase	143.	Vert_HS_TF
106.	Pkinase_C	144.	WD40
107.	Pkinase_Tyr	145.	WW
108.	Pox_A_type_inc	146.	ZZ
109.	Pro_isomerase	147.	adh_short
110.	RBD	148.	bZIP_1
111.	RCC1	149.	bZIP_2
112.	RGS	150.	dNK
113.	RHD	151.	dUTPase
114.	RMMBL	152.	dsrm
115.	RRM_1	153.	efhand
116.	Rap_GAP	154.	tRNA_anti
117.	Ras	155.	ubiquitin
118.	RhoGEF	156.	zf-B_box
119.	Rhodanese	157.	zf-C2H2
120.	Ribosomal_L7Ae	158.	zf-C3HC4
121.	Ribosomal_S4	159.	zf-MYND
122.	S4		

Table S2: Name of exclusive Pfam nuclear domains.

1.	2-Hacid_dh	43.	CDI
2.	2-Hacid_dh_C	44.	CENP-B_N
3.	3_5_exonuc	45.	CHD5
4.	A1pp	46.	CHDCT2
5.	A4_EXTRA	47.	CHDNT
6.	AAA_3	48.	CITED
7.	AAR2	49.	CP2
8.	AARP2CN	50.	CPSF_A
9.	AF-4	51.	CRM1_C
10.	AKAP95	52.	CSE2
11.	APC8	53.	CTF_NFI
12.	APSES	54.	CTNNB1_binding
13.	AP_endonuc_2	55.	CUT
14.	ARID	56.	Caudal_act
15.	AT_hook	57.	CbiA
16.	A_deamin	58.	Cbl_N
17.	AflR	59.	Cbl_N2
18.	Androgen_recep	60.	Cbl_N3
19.	AreA_N	61.	Cdc6_C
20.	Astacin	62.	Cenp-B_dimeris
21.	Aurora-A_bind	63.	CenpB-DNA-bind
22.	B-block_TFIIIC	64.	Chromo
23.	BAF	65.	Chromo_shadow
24.	BAF1_ABF1	66.	Clp1
25.	BAH	67.	Cmyb_C
26.	BDHCT	68.	Copper-fist
27.	BOP1NT	69.	Coprinus_mating
28.	BRCT	70.	Cor1
29.	BRF1	71.	Creb_binding
30.	BSD	72.	Cyclin_C
31.	Basic	73.	Cyclin_N
32.	Beta-trefoil	74.	DBP10CT
33.	Big_2	75.	DDE
34.	Brix	76.	DDT
35.	Bromo_TP	77.	DEAD_2
36.	Bromodomain	78.	DENN
37.	C1_4	79.	DIM1
38.	CAF1	80.	DKCLD
39.	CBF	81.	DMAP1
40.	CBFB_NFYA	82.	DMAP_binding
41.	CBFD_NFYB_HMF	83.	DNA_RNApol_7kD
42.	CDC24	84.	DNA_binding_1
85.	CDC45	130.	DNA_ligase_A_C

86.	DNA_ligase_A_M	131.	Fork_head
87.	DNA_ligase_A_N	132.	Fork_head_N
88.	DNA_methylase	133.	FtsJ
89.	DNA_mis_repair	134.	Fungal_trans
90.	DNA_pol_B	135.	G-patch
91.	DNA_pol_B_exo	136.	GAGA
92.	DNA_pol_E_B	137.	GATA
93.	DNA_pol_delta_4	138.	GATA-N
94.	DP	139.	GCFC
95.	DSHCT	140.	GCR
96.	DTHCT	141.	GLE1
97.	DUF1086	142.	GRAM
98.	DUF1087	143.	GTF2I
99.	DUF1227	144.	GUCT
100.	DUF1518	145.	Gal4_dimer
101.	DUF1546	146.	Gar1
102.	DUF1605	147.	Glyco_hydro_35
103.	DUF1610	148.	GoLoco
104.	DUF1726	149.	HA2
105.	DUF1744	150.	HAND
106.	DUF1777	151.	HAT
107.	DUF592	152.	HCNGP
108.	DUF663	153.	HDAC_interact
109.	DUF699	154.	HH_signal
110.	DUF902	155.	HIRAN
111.	DUF906	156.	HLH
112.	DZF	157.	HMG14_17
113.	Dfp1_Him1_M	158.	HNF-1A_C
114.	Dpy-30	159.	HNF-1B_C
115.	E2F_TDP	160.	HNF-1_N
116.	ELM2	161.	HORMA
117.	ERCC4	162.	HRDC
118.	ETS_PEA3_N	163.	HSA
119.	Ebp2	164.	HSBP1
120.	Ets	165.	HTH_9
121.	F-actin_cap_A	166.	Hairy_orange
122.	FAT	167.	Hint
123.	FATC	168.	Hira
124.	FF	169.	Hist_deacetyl
125.	FHA	170.	Histone
126.	FTZ	171.	Homeobox
127.	FYRC	172.	Hormone_recep
128.	FYRN	173.	Hox9_act
129.	Fibrillarin	174.	IBR
175.	Fip1	220.	INCENP_ARK-bind

176.	IRF	221.	Mog1
177.	Isy1	222.	Mpp10
178.	JmjC	223.	Mre11_DNA_bind
179.	JmjN	224.	MutL_C
180.	Jun	225.	MutS_I
181.	K167R	226.	MutS_II
182.	KAR9	227.	MutS_III
183.	KIX	228.	MutS_IV
184.	KOW	229.	MutS_V
185.	KRAB	230.	Myb_DNA-bind_2
186.	Kdo	231.	Myb_DNA-binding
187.	Ku	232.	Myc-LZ
188.	Ku_C	233.	Myc_N
189.	Ku_N	234.	Myotub-related
190.	Ku_PK_bind	235.	NAC
191.	LAG1-DNAbind	236.	NAP
192.	LEA_4	237.	NCD1
193.	LEM	238.	NCD2
194.	LIM_bind	239.	NGP1NT
195.	LNS2	240.	NIC
196.	LSM	241.	NOG1
197.	La	242.	NOGCT
198.	Linker_histone	243.	NOP5NT
199.	Lipin_N	244.	NOPS
200.	Lsm_interact	245.	NOSIC
201.	M-inducer_phosp	246.	NOT2_3_5
202.	MAT1	247.	NTP_transf_2
203.	MBD	248.	NUC194
204.	MBT	249.	NUP50
205.	MCM	250.	Nab1
206.	MIF4G_like	251.	Nep1
207.	MIF4G_like_2	252.	Nol1_Nop2_Fmu
208.	MOZ_SAS	253.	Nop
209.	MRG	254.	Nop52
210.	MT-A70	255.	Nop53
211.	MYT1	256.	Not1
212.	Mad3_BUB1_II	257.	Not3
213.	Maf_N	258.	Nsp1_C
214.	Mago_nashi	259.	Nuc_rec_co-act
215.	Mak16	260.	Nucleoplasmin
216.	Med5	261.	Nucleopor_Nup85
217.	Menin	262.	Nucleoporin
218.	Methyltransf_1N	263.	Nucleoporin2
219.	Mis12	264.	Nucleoside_tran
265.	Mis6	310.	Nuf2

266.	Nup133	311.	PROCT
267.	Nup133_N	312.	PRP1_N
268.	Nup153	313.	PRP38
269.	Nup84_Nup100	314.	Paf1
270.	OAR	315.	Peptidase_C50
271.	OGG_N	316.	Peptidase_M24
272.	ORC2	317.	Pescadillo_N
273.	ORC3_N	318.	Pirin
274.	ORC6_1	319.	Pirin_C
275.	ORC6_2	320.	Pol_alpha_B_N
276.	Occludin_ELL	321.	Pou
277.	Oest_recep	322.	Pre-SET
278.	P120R	323.	Prefoldin_2
279.	P53	324.	Prog_receptor
280.	P53_TAD	325.	Protamine_3
281.	P53_tetramer	326.	Protamine_P1
282.	P68HR	327.	Protamine_P2
283.	PA26	328.	Prothymosin
284.	PADR1	329.	Prox1
285.	PAH	330.	Prp18
286.	PAP1	331.	Prp19
287.	PAP_RNA-bind	332.	PseudoU_synth_1
288.	PAP_central	333.	PurA
289.	PARP	334.	QLQ
290.	PARP_reg	335.	R3H
291.	PAX	336.	RAG2
292.	PBC	337.	RBB1NT
293.	PC4	338.	RBM1CTR
294.	PCAF_N	339.	RB_A
295.	PCNA_C	340.	RB_B
296.	PCNA_N	341.	RED_C
297.	PD-C2-AF1	342.	RED_N
298.	PHD	343.	RFC1
299.	PI-PLC-X	344.	RFX1_trans_act
300.	PI-PLC-Y	345.	RFX_DNA_binding
301.	PI3_PI4_kinase	346.	RNA_POL_M_15KD
302.	PLC-beta_C	347.	RNA_polI_A14
303.	PLDc	348.	RNA_pol_A_bac
304.	PMC2NT	349.	RNA_pol_I_A49
305.	PNK3P	350.	RNA_pol_L
306.	POP1	351.	RNA_pol_N
307.	POPLD	352.	RNA_pol_Rpa2_4
308.	PPP5	353.	RNA_pol_Rpb1_1
309.	PRO8NT	354.	RNA_pol_Rpb1_2
355.	PROCN	400.	RNA_pol_Rpb1_3

356.	RNA_pol_Rpb1_4	401.	Ribonuclease_3
357.	RNA_pol_Rpb1_5	402.	Rotamase
358.	RNA_pol_Rpb1_6	403.	Rox3
359.	RNA_pol_Rpb1_7	404.	Rtt106
360.	RNA_pol_Rpb1_R	405.	Runt
361.	RNA_pol_Rpb2_1	406.	RunxI
362.	RNA_pol_Rpb2_2	407.	S1
363.	RNA_pol_Rpb2_3	408.	SAM_PNT
364.	RNA_pol_Rpb2_4	409.	SAP
365.	RNA_pol_Rpb2_5	410.	SCA7
366.	RNA_pol_Rpb2_6	411.	SCAN
367.	RNA_pol_Rpb2_7	412.	SCP-1
368.	RNA_pol_Rpb4	413.	SET
369.	RNA_pol_Rpb5_C	414.	SF3b1
370.	RNA_pol_Rpb5_N	415.	SH3BGR
371.	RNA_pol_Rpb6	416.	SIP1
372.	RNA_pol_Rpb7_N	417.	SIR2
373.	RNA_pol_Rpb8	418.	SKIP_SNW
374.	RNA_pol_Rpc34	419.	SLIDE
375.	RNA_pol_Rpc4	420.	SMC_N
376.	RNA_pol_Rpc82	421.	SMC_hinge
377.	RPA_C	422.	SNF2_N
378.	RRM_3	423.	SNF5
379.	RRS1	424.	SPRY
380.	RTC	425.	SPT2
381.	RTC_insert	426.	SRC-1
382.	Rad10	427.	SRF-TF
383.	Rad17	428.	SRP40_C
384.	Rad21_Rec8	429.	SSDP
385.	Rad21_Rec8_N	430.	SSrecog
386.	Rad4	431.	STAG
387.	Rad51	432.	STAT_alpha
388.	Rad54_N	433.	STAT_bind
389.	Rad9_Rad53_bind	434.	STAT_int
390.	Ran_BP1	435.	STE
391.	Rap1-DNA-bind	436.	SURF6
392.	Rb_C	437.	SWIB
393.	RecQ5	438.	SWIRM
394.	Red1	439.	Sad1_UNC
395.	Rep-A_N	440.	Sec20
396.	RepA_N	441.	Sedlin_N
397.	Rep_fac-A_3	442.	Serine_rich
398.	Rep_fac-A_C	443.	Sina
399.	ResIII	444.	Ski_Sno
445.	Response_reg	490.	Skp1

446.	Skp1_POZ	491.	TP2
447.	Snf7	492.	TP6A_N
448.	Sof1	493.	TPX2
449.	Sp100	494.	TRF
450.	Spb1_C	495.	Tcp11
451.	Spc97_Spc98	496.	Telo_bind
452.	Spot_14	497.	Tfb2
453.	Spt4	498.	Tfb4
454.	Ssl1	499.	ThiF
455.	Supt5	500.	Tis11B_N
456.	Surp	501.	Translin
457.	T-box	502.	Transposase_5
458.	TAF	503.	TruB_N
459.	TAF4	504.	U-box
460.	TAFH	505.	UBACT
461.	TAFII28	506.	UBA_2
462.	TAFII55_N	507.	UDG
463.	TAP_C	508.	UME
464.	TBP	509.	Ustilago_mating
465.	TBP-binding	510.	UvrD-helicase
466.	TBPIP	511.	Vg_Tdu
467.	TCH	512.	WGR
468.	TEA	513.	WT1
469.	TEBP_beta	514.	Wos2
470.	TFIIA	515.	XPA_C
471.	TFIIA_gamma_C	516.	XPA_N
472.	TFIIA_gamma_N	517.	XPC-binding
473.	TFIIB	518.	XPG_I
474.	TFIIB_Zn_Ribbon	519.	XPG_N
475.	TFIID-18kDa	520.	XRN_N
476.	TFIID-31kDa	521.	Xpo1
477.	TFIID_20kDa	522.	YEATS
478.	TFIID_30kDa	523.	YL1
479.	TFIID_90kDa	524.	YL1_C
480.	TFIIE_alpha	525.	Zfx_Zfy_act
481.	TFIIE_beta	526.	Zn_clus
482.	TFIIF_alpha	527.	Zw10
483.	TFIIF_beta	528.	bZIP_Maf
484.	TFIIH_BTF_p62_N	529.	c-SKI_SMAD_bind
485.	TFIIS	530.	dDENN
486.	TFIIS_C	531.	eIF-3_zeta
487.	TFIIS_M	532.	efhand_like
488.	TF_AP-2	533.	mRNA_cap_C
489.	TLE_N	534.	mRNA_cap_enzyme
535.	TP1	547.	mRNA_triPase

536.	pKID	548.	zf-DNA_Pol
537.	uDENN	549.	zf-GRF
538.	z-alpha	550.	zf-LYAR
539.	zf-C2HC	551.	zf-MIZ
540.	zf-C4	552.	zf-NF-X1
541.	zf-C4_C	553.	zf-PARP
542.	zf-C5HC2	554.	zf-RNPHF
543.	zf-CCCH	555.	zf-RanBP
544.	zf-CCHC	556.	zf-TAZ
545.	zf-CXXC	557.	zf-U1
546.	zf-DBF	558.	zf-UBR

Table S3: Exclusive non-nuclear Pfam domains.

1.	14-3-3	43.	ATE_N
2.	2-oxoacid_dh	44.	ATP-cone
3.	2OG-FeII_Oxy	45.	ATP-grasp_2
4.	3-HAO	46.	ATP-gua_Ptrans
5.	3Beta_HSD	47.	ATP-gua_PtransN
6.	3HCDH	48.	ATP-synt
7.	3HCDH_N	49.	ATP-synt_10
8.	4HBT	50.	ATP-synt_C
9.	5-FTHF_cyc-lig	51.	ATP-synt_DE
10.	5_nucleotid	52.	ATP-synt_DE_N
11.	5_nucleotid_C	53.	ATP-synt_Eps
12.	60KD_IMP	54.	ATP-synt_ab
13.	7tm_1	55.	ATP-synt_ab_C
14.	A1_Propeptide	56.	ATP-synt_ab_N
15.	AAA_2	57.	ATP11
16.	AA_kinase	58.	ATP12
17.	ABC1	59.	ATX_III
18.	ABC_membrane	60.	Abhydro_lipase
19.	ABC_membrane_2	61.	Abhydrolase_1
20.	ABC_tran	62.	Abi
21.	ACBP	63.	Acetyltransf_2
22.	ACC_central	64.	Acid_phosphat_A
23.	ACOX	65.	Aconitase
24.	ACT	66.	Aconitase_C
25.	ADAM_spacer1	67.	Acp26Ab
26.	ADH_N	68.	Acyl-CoA_dh_1
27.	ADH_zinc_N	69.	Acyl-CoA_dh_2
28.	ADK	70.	Acyl-CoA_dh_M
29.	ADK_lid	71.	Acyl-CoA_dh_N
30.	AHSP	72.	Acyl_CoA_thio
31.	AIRS	73.	Acyltransferase
32.	AIRS_C	74.	Adaptin_N
33.	ALG3	75.	Adenylsucc_synt
34.	ALO	76.	AdoHcyase
35.	AMP-binding	77.	AdoHcyase_NAD
36.	ANATO	78.	Adrenomedullin
37.	ANP	79.	Agenet
38.	ANTH	80.	Agouti
39.	APG5	81.	AhpC-TSA
40.	APH	82.	AlaDh_PNT_C
41.	ART	83.	AlaDh_PNT_N
42.	ATE_C	84.	Ald_Xan_dh_C
85.	Ald_Xan_dh_C2	130.	Asp_Arg_Hydrox

86.	Aldedh	131.	Asparaginase
87.	Aldo_ket_red	132.	Atracotoxin
88.	Alg6_Alg8	133.	Attacin_C
89.	Alpha-amylase	134.	Attacin_N
90.	Alpha-amylase_C	135.	Avidin
91.	Alpha-mann_mid	136.	Axin_b-cat_bind
92.	Alpha_adaptinC2	137.	B12-binding
93.	Amelin	138.	B12-binding_2
94.	Amelogenin	139.	B3_4
95.	Amidase	140.	B5
96.	Amidohydro_2	141.	BAAT_C
97.	Amino_oxidase	142.	BAR
98.	Aminotran_1_2	143.	BCMA-Tall_bind
99.	Aminotran_3	144.	BCS1_N
100.	Aminotran_4	145.	BDS_I_II
101.	Aminotran_5	146.	BNIP3
102.	An_peroxidase	147.	BNR
103.	Anemone_cytotox	148.	BPL_LipA_LipB
104.	Annexin	149.	BRICHOS
105.	Anp1	150.	BSP_II
106.	Anticodon_1	151.	BTG
107.	Antimicrobial10	152.	BTK
108.	Antimicrobial12	153.	B_lectin
109.	Antimicrobial_1	154.	Band_41
110.	Antimicrobial_2	155.	Band_7
111.	Antimicrobial_3	156.	Bap31
112.	Antimicrobial_4	157.	Bclx_interact
113.	Antimicrobial_6	158.	Bile_Hydr_Trans
114.	Antimicrobial_7	159.	Biliv-reduc_cat
115.	Antistasin	160.	Bim_N
116.	Apo-CII	161.	Biotin_carb_C
117.	Apo-CIII	162.	Biotin_lipoyl
118.	ApoA-II	163.	Bombinin
119.	ApoC-I	164.	Bradykinin
120.	ApoL	165.	Branch
121.	ApoLp-III	166.	Brevenin
122.	Apolipoprotein	167.	C1-set
123.	Arfaptin	168.	C1q
124.	ArgJ	169.	C5-epim_C
125.	Arg_tRNA_synt_N	170.	CAP_GLY
126.	Arginase	171.	CBM_1
127.	Arylesterase	172.	CBM_10
128.	Asp	173.	CBM_14
129.	Asp-B-Hydro_N	174.	CBM_20
175.	CDC37_C	220.	CXCXC

176.	CDC37_M	221.	Caenor_Her-1
177.	CDC37_N	222.	Calc_CGRP_IAPP
178.	CDC48_2	223.	Calpain_III
179.	CDC48_N	224.	Calreticulin
180.	CDP-OH_P_transf	225.	Calx-beta
181.	CH	226.	Carb_anhydrase
182.	CHCH	227.	Carboxyl_trans
183.	CHRD	228.	Carn_acyltransf
184.	CI-B14_5a	229.	Casein
185.	CIA30	230.	Casein_kappa
186.	CIDE-N	231.	Catalase
187.	CLP_protease	232.	Cation_ATPase_C
188.	CNH	233.	Cation_ATPase_N
189.	CNTF	234.	Cation_efflux
190.	CN_hydrolase	235.	Cecropin
191.	COG4	236.	Cerato-platanin
192.	COLFI	237.	Chitin_synth_1
193.	COPI_C	238.	Chitin_synth_1N
194.	COX1	239.	Chitin_synth_2
195.	COX15-CtaA	240.	Choline_kin_N
196.	COX17	241.	Choline_kinase
197.	COX2	242.	Citrate_synt
198.	COX2_TM	243.	Clat_adaptor_s
199.	COX3	244.	Clathrin
200.	COX4	245.	Clathrin-link
201.	COX5A	246.	Clathrin_lg_ch
202.	COX5B	247.	Clathrin_propel
203.	COX6A	248.	Clavanin
204.	COX6C	249.	Clusterin
205.	COX7B	250.	CoA_binding
206.	COX7C	251.	CoA_trans
207.	COX7a	252.	Coagulin
208.	COX8	253.	Coatamer_beta_C
209.	CO_deh_flav_C	254.	Coatomer_E
210.	COesterase	255.	Coatomer_WDAD
211.	CPSase_L_D2	256.	Cobalamin_bind
212.	CPSase_L_D3	257.	Cofilin_ADF
213.	CPSase_L_chain	258.	Coleoptericin
214.	CPSase_sm_chain	259.	Colipase
215.	CRF	260.	Colipase_C
216.	CSF-1	261.	Complex1_49kDa
217.	CTP_transf_1	262.	Complex1_51K
218.	CTP_transf_2	263.	Complex1_LYR
219.	CUE	264.	Conotoxin
265.	Coprogen_oxidas	310.	DUF1000

266.	Coq4	311.	DUF1081
267.	CorA	312.	DUF1787
268.	Cornifin	313.	DUF1866
269.	Cpn10	314.	DUF1903
270.	Cpn60_TCP1	315.	DUF1929
271.	Crisp	316.	DUF1943
272.	CtaG_Cox11	317.	DUF1981
273.	Cu-oxidase	318.	DUF1986
274.	Cu-oxidase_2	319.	DUF290
275.	Cu-oxidase_3	320.	DUF619
276.	Cu_amine_oxid	321.	Dak1
277.	Cu_amine_oxidN2	322.	Dak2
278.	Cu_amine_oxidN3	323.	Dala_Dala_lig_C
279.	Cutinase	324.	Death
280.	CybS	325.	Defensin_1
281.	Cys_Met_Meta_PP	326.	Defensin_2
282.	Cys_knot	327.	Defensin_3
283.	Cys_rich_FGFR	328.	Defensin_beta
284.	Cystatin	329.	Defensin_propep
285.	Cyt-b5	330.	Dickkopf_N
286.	Cyto_heme_lyase	331.	Dishevelled
287.	Cytochrom_C	332.	Dor1
288.	Cytochrom_C1	333.	Drf_GBD
289.	DAG1	334.	Dynamitin
290.	DALR_1	335.	Dynein_heavy
291.	DAO	336.	Dynein_light
292.	DDOST_48kD	337.	E1-E2_ATPase
293.	DED	338.	E1_DerP2_DerF2
294.	DEP	339.	E1_dh
295.	DFF-C	340.	E3_binding
296.	DHC_N1	341.	EBP
297.	DHC_N2	342.	ECH
298.	DHHA1	343.	ECM1
299.	DHO_dh	344.	EF_TS
300.	DHQ_synthase	345.	EGF
301.	DHquinase_I	346.	EGF-like_subdom
302.	DIL	347.	EGF_2
303.	DIM	348.	EGF_CA
304.	DIX	349.	ELFV_dehydrog
305.	DNA_pol_A	350.	ELFV_dehydrog_N
306.	DOPA_dioxygen	351.	ELO
307.	DPM2	352.	EMI
308.	DPM3	353.	EMP24_GP25L
309.	DSBA	354.	ENTH
355.	EPO_TPO	400.	FMN_dh

356.	EPSP_synthase	401.	FOLN
357.	ERG2_Sigma1R	402.	FTCD
358.	ERM	403.	FTCD_C
359.	ERp29	404.	FTCD_N
360.	ERp29_N	405.	FTHFS
361.	ETC_C1_NDUFA4	406.	FYVE
362.	ETC_C1_NDUFA5	407.	F_actin_bind
363.	ETF	408.	Fasciclin
364.	ETF_QO	409.	Fer2
365.	ETF_alpha	410.	Fer2_2
366.	EXTL2	411.	Fer4
367.	E_raikovi_mat	412.	Ferritin
368.	Ectatomin	413.	Ferrochelatase
369.	Elicitin	414.	Fibrinogen_C
370.	Endonuclease_5	415.	Fibroin_P25
371.	Endonuclease_NS	416.	Filament_head
372.	Endosulfine	417.	Filamin
373.	Endothelin	418.	Flavodoxin_1
374.	Enolase_C	419.	Flavodoxin_2
375.	Enolase_N	420.	Flavoprotein
376.	Ependymin	421.	Focal_AT
377.	Epimerase	422.	Folate_rec
378.	Esterase	423.	Formyl_trans_C
379.	Euplotes_phero	424.	Formyl_trans_N
380.	Evr1_Alr	425.	Frataxin_Cyay
381.	Exonuc_X-T	426.	Fringe
382.	Exostosin	427.	FtsH_ext
383.	Extensin_1	428.	Fucokinase
384.	F5_F8_type_C	429.	G-gamma
385.	FAD-oxidase_C	430.	G6PD_C
386.	FAD_binding_1	431.	G6PD_N
387.	FAD_binding_2	432.	GAS2
388.	FAD_binding_3	433.	GAT
389.	FAD_binding_4	434.	GATase
390.	FAD_binding_5	435.	GBP_repeat
391.	FAD_binding_6	436.	GCC2_GCC3
392.	FARP	437.	GCV_H
393.	FA_desaturase	438.	GCV_T
394.	FCH	439.	GCV_T_C
395.	FDX-ACB	440.	GDA1_CD39
396.	FG-GAP	441.	GDC-P
397.	FGF	442.	GDI
398.	FGGY_C	443.	GFO_IDH_MocA
399.	FGGY_N	444.	GHMP_kinases_C
445.	GHMP_kinases_N	490.	Glyco_hydro_38C

446.	GLTP	491.	Glyco_hydro_45
447.	GMAP	492.	Glyco_hydro_47
448.	GMC_oxred_C	493.	Glyco_hydro_49
449.	GMC_oxred_N	494.	Glyco_hydro_6
450.	GMP_synt_C	495.	Glyco_hydro_61
451.	GNT-I	496.	Glyco_hydro_62
452.	GPI2	497.	Glyco_hydro_63
453.	GRIM-19	498.	Glyco_hydro_67C
454.	GRIP	499.	Glyco_hydro_67M
455.	GSHPx	500.	Glyco_hydro_67N
456.	GST_C	501.	Glyco_hydro_7
457.	GST_N	502.	Glyco_transf_15
458.	GTPase_binding	503.	Glyco_transf_17
459.	Gaa1	504.	Glyco_transf_22
460.	Gal_Lectin	505.	Glyco_transf_29
461.	Galactosyl_T	506.	Glyco_transf_34
462.	Galactosyl_T_2	507.	Glyco_transf_43
463.	Galanin	508.	Glycos_transf_1
464.	Gamma-COP	509.	Glycos_transf_2
465.	Gamma-thionin	510.	Glycos_transf_4
466.	Gastrin	511.	Gp_dh_C
467.	GatB	512.	Gp_dh_N
468.	GatB_N	513.	GrpE
469.	GatB_Yqey	514.	Guanylate_cyc
470.	Gelsolin	515.	Guanylate_kin
471.	Gla	516.	Guanylin
472.	Gln-synt_C	517.	HEAT_PBS
473.	Gln-synt_N	518.	HELP
474.	Globin	519.	HGTP_anticodon
475.	Glucosamine_iso	520.	HIT
476.	Glutaminase	521.	HMA
477.	Glutaredoxin	522.	HMG-CoA_red
478.	Gly_transf_sug	523.	HMGL-like
479.	Glyco_hydro_10	524.	HMG_CoA_synt_C
480.	Glyco_hydro_11	525.	HMG_CoA_synt_N
481.	Glyco_hydro_12	526.	HNOB
482.	Glyco_hydro_15	527.	HNOBA
483.	Glyco_hydro_16	528.	HR1
484.	Glyco_hydro_18	529.	HS2ST
485.	Glyco_hydro_28	530.	HSP20
486.	Glyco_hydro_31	531.	HSP70
487.	Glyco_hydro_32C	532.	HSP90
488.	Glyco_hydro_32N	533.	Hamartin
489.	Glyco_hydro_38	534.	He_PIG
535.	Hemocyanin_C	580.	I_LWEQ

536.	Hemocyanin_M	581.	IlvC
537.	Hemocyanin_N	582.	IlvN
538.	Hemopexin	583.	Img2
539.	Hepcidin	584.	Inhibitor_I29
540.	Hexokinase_1	585.	Inos-1-P_synth
541.	Hexokinase_2	586.	Inositol_P
542.	Hirudin	587.	Ins145_P3_rec
543.	His_binding	588.	Insulin
544.	Hormone_1	589.	Interferon
545.	Hormone_2	590.	Involucrin
546.	Hormone_3	591.	Involucrin2
547.	Hormone_4	592.	Ion_trans
548.	Hormone_5	593.	Iso_dh
549.	Hormone_6	594.	JHBP
550.	Hydrophobin	595.	KR
551.	Hydrophobin_2	596.	KRE9
552.	IATP	597.	Kazal_1
553.	IBB	598.	Kazal_2
554.	ICL	599.	Kringle
555.	ICMT	600.	L-fibroin
556.	ICln_channel	601.	L51_S25_CI-B8
557.	IF2_N	602.	LAG1
558.	IFN-gamma	603.	LEAP-2
559.	IGF2_C	604.	LETM1
560.	IGFBP	605.	LIP
561.	IKI3	606.	LMWPc
562.	IL1	607.	LON
563.	IL10	608.	LRRNT
564.	IL11	609.	Laminin_B
565.	IL12	610.	Laminin_EGF
566.	IL13	611.	Laminin_G_1
567.	IL15	612.	Laminin_G_2
568.	IL17	613.	Laminin_I
569.	IL2	614.	Laminin_II
570.	IL3	615.	Laminin_N
571.	IL4	616.	Lamprin
572.	IL4Ra_N	617.	Ldh_1_C
573.	IL5	618.	Ldh_1_N
574.	IL6	619.	Ldl_recept_a
575.	IL7	620.	Lectin_C
576.	IL8	621.	Lectin_leg-like
577.	ILVD_EDD	622.	Leptin
578.	IP_trans	623.	Leuk-A4-hydro_C
579.	IQ	624.	Ligase_CoA
625.	Lipase	670.	Mo25

626.	Lipase_3	671.	MoCF_biosynth
627.	Lipocalin	672.	MoeA_C
628.	Lipocalin_2	673.	MoeA_N
629.	Lipoprotein_11	674.	Moricin
630.	Lipoxygenase	675.	Motile_Sperm
631.	Lon_C	676.	Motilin_assoc
632.	Lys	677.	Motilin_ghrelin
633.	Lysyl_oxidase	678.	Mpv17_PMP22
634.	M20_dimer	679.	Mt_ATP-synt_B
635.	MACPF	680.	Mtap_PNP
636.	MAGE	681.	Mtc
637.	MAGP	682.	Mu-conotoxin
638.	MAGSP	683.	Muskelin_N
639.	MAM	684.	MyTH4
640.	MAM33	685.	Myelin_MBP
641.	MANEC	686.	Myosin_N
642.	MAS20	687.	Myosin_TH1
643.	MBA1	688.	Myosin_head
644.	MBOAT	689.	NACHT
645.	MFAP1_C	690.	NADH_dehy_S2_C
646.	MFS_1	691.	NADH_oxidored
647.	MGAT2	692.	NAD_Gly3P_dh_C
648.	MGC-24	693.	NAD_Gly3P_dh_N
649.	MGS	694.	NAD_binding_1
650.	MHC_I	695.	NAD_binding_2
651.	MHC_II_alpha	696.	NAD_binding_4
652.	MHC_II_beta	697.	NAD_binding_5
653.	MIF	698.	NAPRTase
654.	MIP	699.	NB-ARC
655.	MIR	700.	NC
656.	MM_CoA_mutase	701.	NDK
657.	MORN	702.	NDUFA12
658.	MRJP	703.	NDUF_B12
659.	Malic_M	704.	NDUF_B4
660.	MaoC_dehydratas	705.	NDUF_B7
661.	Melittin	706.	NDUF_B8
662.	Met_synt_B12	707.	NDUF_C2
663.	Metallothionein	708.	NGF
664.	Methyltransf_11	709.	NIF3
665.	Methyltransf_12	710.	NMT
666.	Microtub_assoc	711.	NMT_C
667.	Mit_preoteolip	712.	NMU
668.	Mito_carr	713.	NNMT_PNMT_TEMT
669.	Mo-co_dimer	714.	NSF
715.	NTR	760.	PDEase_I

716.	NUDIX	761.	PDEase_I_N
717.	Na_Ca_ex	762.	PDGF
718.	Na_H_Exchanger	763.	PEPCK
719.	Neur_chan_LBD	764.	PET122
720.	Neurokinin_B	765.	PEX11
721.	Neuromodulin	766.	PGI
722.	Nexin_C	767.	PGK
723.	Nitrophorin	768.	PGM_PMM_I
724.	Noggin	769.	PGM_PMM_II
725.	Nramp	770.	PGM_PMM_III
726.	Nuc_sug_transp	771.	PGM_PMM_IV
727.	OCD_Mu_crystall	772.	PG_binding_1
728.	OLF	773.	PIG-F
729.	OSCP	774.	PIG-L
730.	OST3_OST6	775.	PIGA
731.	OTCace	776.	PIR
732.	OTCace_N	777.	PLA2G12
733.	OTU	778.	PLA2_B
734.	Olfactory_mark	779.	PLA2_inh
735.	Omega-toxin	780.	PLAC
736.	Opiods_neuropep	781.	PLAT
737.	Orexin	782.	PMI_typeI
738.	Osmo_CC	783.	PMM
739.	Osteopontin	784.	PMP1_2
740.	Oxidored_molyb	785.	PMT
741.	Oxidored_q1	786.	PNTB
742.	Oxidored_q3	787.	PP-binding
743.	Oxysterol_BP	788.	PPR
744.	P-mevalo_kinase	789.	PQQ
745.	P4Ha_N	790.	PSP94
746.	PAF-AH_p_II	791.	PS_Dcarbxylase
747.	PALP	792.	PUF
748.	PAM2	793.	PXA
749.	PAN_1	794.	PYC_OADA
750.	PAP2	795.	P_proprotein
751.	PAPS_reduct	796.	Pam16
752.	PAP_assoc	797.	Parathyroid
753.	PBD	798.	Pardaxin
754.	PBP	799.	Patatin
755.	PBP_GOBP	800.	Penaeidin
756.	PCI	801.	Pentaxin
757.	PCMT	802.	Pep3_Vps18
758.	PCRF	803.	Pep_M12B_propep
759.	PDCD9	804.	Peptidase_A22B
805.	Peptidase_C1	850.	Pro-NT_NN

806.	Peptidase_C12	851.	Pro_dh
807.	Peptidase_C14	852.	Prokineticin
808.	Peptidase_C1_2	853.	Pterin_bind
809.	Peptidase_C2	854.	Pyr_redox
810.	Peptidase_C26	855.	Pyr_redox_2
811.	Peptidase_M10	856.	Pyr_redox_dim
812.	Peptidase_M14	857.	Pyrophosphatase
813.	Peptidase_M16	858.	RF-1
814.	Peptidase_M16_C	859.	RIH_assoc
815.	Peptidase_M17	860.	RINT1_TIP1
816.	Peptidase_M17_N	861.	RNA_pol
817.	Peptidase_M18	862.	RNB
818.	Peptidase_M2	863.	RNase_U2
819.	Peptidase_M20	864.	RPM2
820.	Peptidase_M28	865.	RRF
821.	Peptidase_M3	866.	RYDR_ITPR
822.	Peptidase_M41	867.	Radical_SAM
823.	Peptidase_M43	868.	RasGAP
824.	Peptidase_M48	869.	Redoxin
825.	Peptidase_M49	870.	Reeler
826.	Peptidase_S10	871.	Reprolysin
827.	Peptidase_S8	872.	Rer1
828.	Peptidase_S9	873.	Resistin
829.	Peptidase_S9_N	874.	Reticulon
830.	Pericardin_rpt	875.	RhoGAP
831.	Peroxin-13_N	876.	Rho_GDI
832.	Peroxin-3	877.	Rhomboid
833.	Pex14_N	878.	Rib_recp_KP_reg
834.	Pex16	879.	Ribonuc_2-5A
835.	Pex19	880.	Ribonuc_L-PSP
836.	Pex2_Pex12	881.	Ribonuc_red_lgC
837.	Phe_hydrox_dim	882.	Ribonuc_red_lgN
838.	Phosphodiest	883.	Ribonuc_red_sm
839.	Phosphoesterase	884.	Ribophorin_I
840.	Phospholip_A2_1	885.	Ribophorin_II
841.	Phospholip_A2_2	886.	Ribosomal_L1
842.	PhyH	887.	Ribosomal_L10e
843.	Plectin	888.	Ribosomal_L11
844.	Porin_3	889.	Ribosomal_L11_N
845.	Prefoldin	890.	Ribosomal_L12
846.	Prenyltransf	891.	Ribosomal_L14
847.	Presenilin	892.	Ribosomal_L16
848.	Preseq_ALAS	893.	Ribosomal_L17
849.	Pribosyltran	894.	Ribosomal_L18e
895.	Ribosomal_L18p	940.	SCP

896.	Ribosomal_L2	941.	SCP2
897.	Ribosomal_L23	942.	SEA
898.	Ribosomal_L27	943.	SGL
899.	Ribosomal_L28	944.	SH3BP5
900.	Ribosomal_L2_C	945.	SHMT
901.	Ribosomal_L3	946.	SHR3_chaperone
902.	Ribosomal_L32p	947.	SKI
903.	Ribosomal_L36	948.	SKN1
904.	Ribosomal_L44	949.	SNAP-25
905.	Ribosomal_L5	950.	SNARE
906.	Ribosomal_L5_C	951.	SOCS_box
907.	Ribosomal_L6	952.	SP_C-Propep
908.	Ribosomal_L9_N	953.	SQS_PSY
909.	Ribosomal_MRP8	954.	SRCR
910.	Ribosomal_S10	955.	SRP-alpha_N
911.	Ribosomal_S11	956.	SRP14
912.	Ribosomal_S12	957.	SRP19
913.	Ribosomal_S13	958.	SRP54
914.	Ribosomal_S14	959.	SRP54_N
915.	Ribosomal_S15	960.	SRP72
916.	Ribosomal_S16	961.	SRP9
917.	Ribosomal_S18	962.	SRP_SPB
918.	Ribosomal_S19	963.	SRX
919.	Ribosomal_S2	964.	SSB
920.	Ribosomal_S3Ae	965.	START
921.	Ribosomal_S3_C	966.	SUN
922.	Ribosomal_S3_N	967.	SURF1
923.	Ribosomal_S5	968.	SURF4
924.	Ribosomal_S6	969.	S_100
925.	Ribosomal_S7	970.	SapA
926.	Ribosomal_S8	971.	SapB_1
927.	Ribosomal_S9	972.	SapB_2
928.	Ricin_B_lectin	973.	Sdh_cyt
929.	Rieske	974.	Sds3
930.	RnaseA	975.	Sec1
931.	RrnaAD	976.	Sec10
932.	S-AdoMet_synt_C	977.	Sec23_BS
933.	S-AdoMet_synt_M	978.	Sec23_helical
934.	S-AdoMet_synt_N	979.	Sec23_trunk
935.	S-methyl_trans	980.	Sec34
936.	S1-P1_nuclease	981.	Sec6
937.	S10_plectin	982.	Sec62
938.	SBP56	983.	Sec7
939.	SCO1-SenC	984.	SecY
985.	SelP_C	1030.	TB

986.	SelP_N	1031.	TCTP
987.	Sema	1032.	TFR_dimer
988.	Semenogelin	1033.	TGF_beta
989.	Semialdhyde_dh	1034.	TGFb_propeptide
990.	Semialdhyde_dhC	1035.	TGS
991.	Sep15_SelM	1036.	THF_DHG_CYH
992.	Serendipity_A	1037.	THF_DHG_CYH_C
993.	Serinc	1038.	TIL
994.	Serpin	1039.	TIMP
995.	Serum_albumin	1040.	TIR
996.	Seryl_tRNA_N	1041.	TK
997.	Sex_peptide	1042.	TNF
998.	ShK	1043.	TNFR_c6
999.	Shikimate_DH	1044.	TPMT
1000.	Shikimate_dh_N	1045.	TPP_enzyme_C
1001.	Sod_Cu	1046.	TPP_enzyme_M
1002.	Sod_Fe_C	1047.	TPP_enzyme_N
1003.	Sod_Fe_N	1048.	TPT
1004.	Somatomedin_B	1049.	TRAM1
1005.	Somatostatin	1050.	TRAP-delta
1006.	Spectrin	1051.	TRAP-gamma
1007.	SpoU_methylase	1052.	TRAPP_Bet3
1008.	SpoU_sub_bind	1053.	TRAP_alpha
1009.	Spond_N	1054.	TRAP_beta
1010.	Spp-24	1055.	TROVE
1011.	Sprouty	1056.	TSP_1
1012.	Stanniocalcin	1057.	TSP_3
1013.	Statherin	1058.	Tannase
1014.	Stathmin	1059.	TauD
1015.	Sterol_desat	1060.	Terpene_synth_C
1016.	Striatin	1061.	Thiolase_C
1017.	Subtilisin_N	1062.	Thiolase_N
1018.	Succ_DH_flav_C	1063.	Thioredoxin
1019.	Sulfatase	1064.	Thr_dehydrat_C
1020.	Sulfotransfer_1	1065.	Thymosin
1021.	SurE	1066.	Thyroglobulin_1
1022.	Surfac_D-trimer	1067.	Tim17
1023.	Sushi	1068.	Tim44
1024.	Sushi_2	1069.	Tom22
1025.	Sybindin	1070.	Tom7
1026.	Syja_N	1071.	Topoisom_I
1027.	Synaptobrevin	1072.	Topoisom_I_N
1028.	Syntaxin	1073.	Torsin
1029.	T4_deiodinase	1074.	Toxin_1
1075.	Toxin_11	1120.	UCR_hinge

1076.	Toxin_12	1121.	UDP-g_GGTase
1077.	Toxin_13	1122.	UDPGP
1078.	Toxin_14	1123.	UPAR_LY6
1079.	Toxin_16	1124.	UPF0224
1080.	Toxin_2	1125.	URO-D
1081.	Toxin_20	1126.	UbiA
1082.	Toxin_21	1127.	Ubiq-Cytc-red_N
1083.	Toxin_23	1128.	Ubiq_cyt_C_chap
1084.	Toxin_24	1129.	UcrQ
1085.	Toxin_25	1130.	Upf2
1086.	Toxin_3	1131.	Uricase
1087.	Toxin_30	1132.	Uteroglobin
1088.	Toxin_32	1133.	V-ATPase_G
1089.	Toxin_33	1134.	V-SNARE
1090.	Toxin_34	1135.	V-set
1091.	Toxin_4	1136.	VAR1
1092.	Toxin_5	1137.	VHS
1093.	Toxin_6	1138.	VPS9
1094.	Toxin_7	1139.	VSP
1095.	Toxin_8	1140.	VWA
1096.	Toxin_9	1141.	VWC
1097.	Transferrin	1142.	VWD
1098.	Transglut_C	1143.	Vault
1099.	Transglut_N	1144.	Vinculin
1100.	Transglut_core	1145.	VitD-bind_III
1101.	Transket_pyr	1146.	Vitellogenin_N
1102.	Transketolase_C	1147.	Vps16_C
1103.	Transketolase_N	1148.	Vps16_N
1104.	Transthyretin	1149.	Vps26
1105.	Trefoil	1150.	Vps35
1106.	Trehalase	1151.	Vps5
1107.	Trehalase_Ca-bi	1152.	WAP
1108.	Triabin	1153.	WH2
1109.	Tropomyosin	1154.	WHEP-TRS
1110.	Trypsin	1155.	WIF
1111.	Tsg	1156.	Xlink
1112.	TspO_MBR	1157.	Y_phosphatase
1113.	Tub	1158.	Yeast-kill-tox
1114.	Tuberin	1159.	ZU5
1115.	UAA	1160.	Zip
1116.	UCR_14kD	1161.	Zona_pellucida
1117.	UCR_6-4kD	1162.	aPHC
1118.	UCR_TM	1163.	eRF1_1
1119.	UCR_UQCRX_QCR9	1164.	eRF1_2
1165.	eRF1_3	1182.	tRNA-synt_1c_C

1166.	efhand_1	1183.	tRNA-synt_1d
1167.	efhand_2	1184.	tRNA-synt_1e
1168.	efhand_Ca_insen	1185.	tRNA-synt_1g
1169.	fn1	1186.	tRNA-synt_2
1170.	fn2	1187.	tRNA-synt_2b
1171.	fn3	1188.	tRNA-synt_2c
1172.	ig	1189.	tRNA-synt_2d
1173.	mTERF	1190.	tRNA_SAD
1174.	malic	1191.	tRNA_synt_1c_R1
1175.	p450	1192.	tRNA_synt_1c_R2
1176.	p47_phox_C	1193.	wnt
1177.	peroxidase	1194.	zf-A20
1178.	polyprenyl_synt	1195.	zf-Sec23_Sec24
1179.	tRNA-synt_1	1196.	zf-TRAF
1180.	tRNA-synt_1b	1197.	zf-Tim10_DDP
1181.	tRNA-synt_1c		

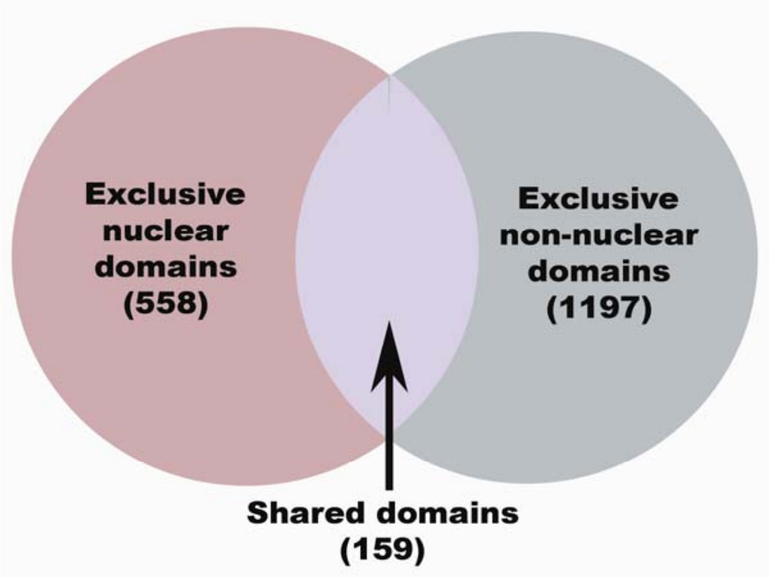

Figure S1: Distribution in exclusive nuclear, non-nuclear and shared domains in NucPfam database.

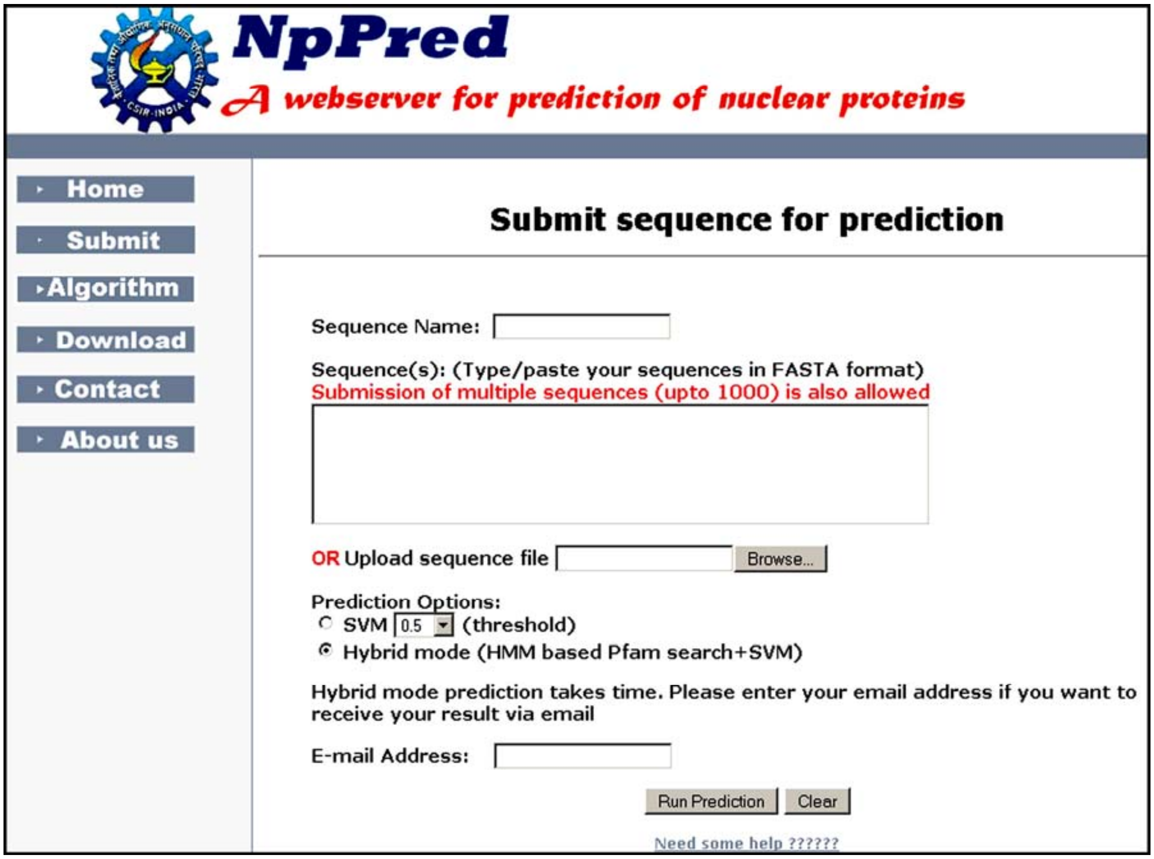

Figure S2: Submission page of NpPred webserver.

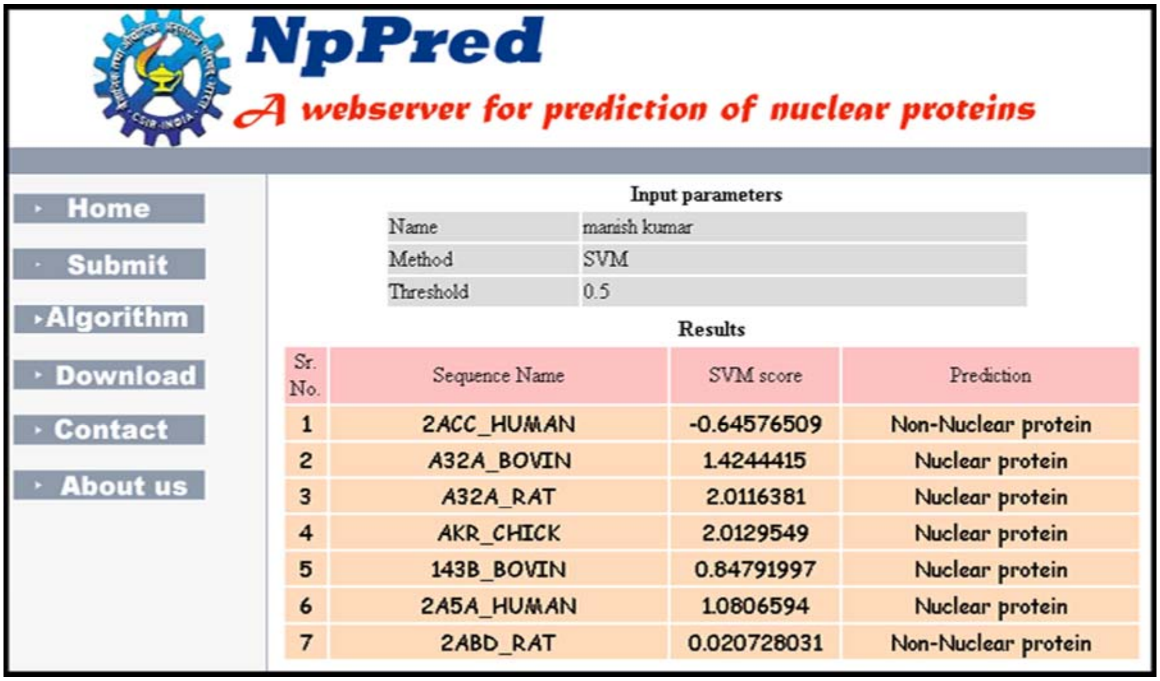

Figure S3: Prediction result of NpPred with SVM approach.

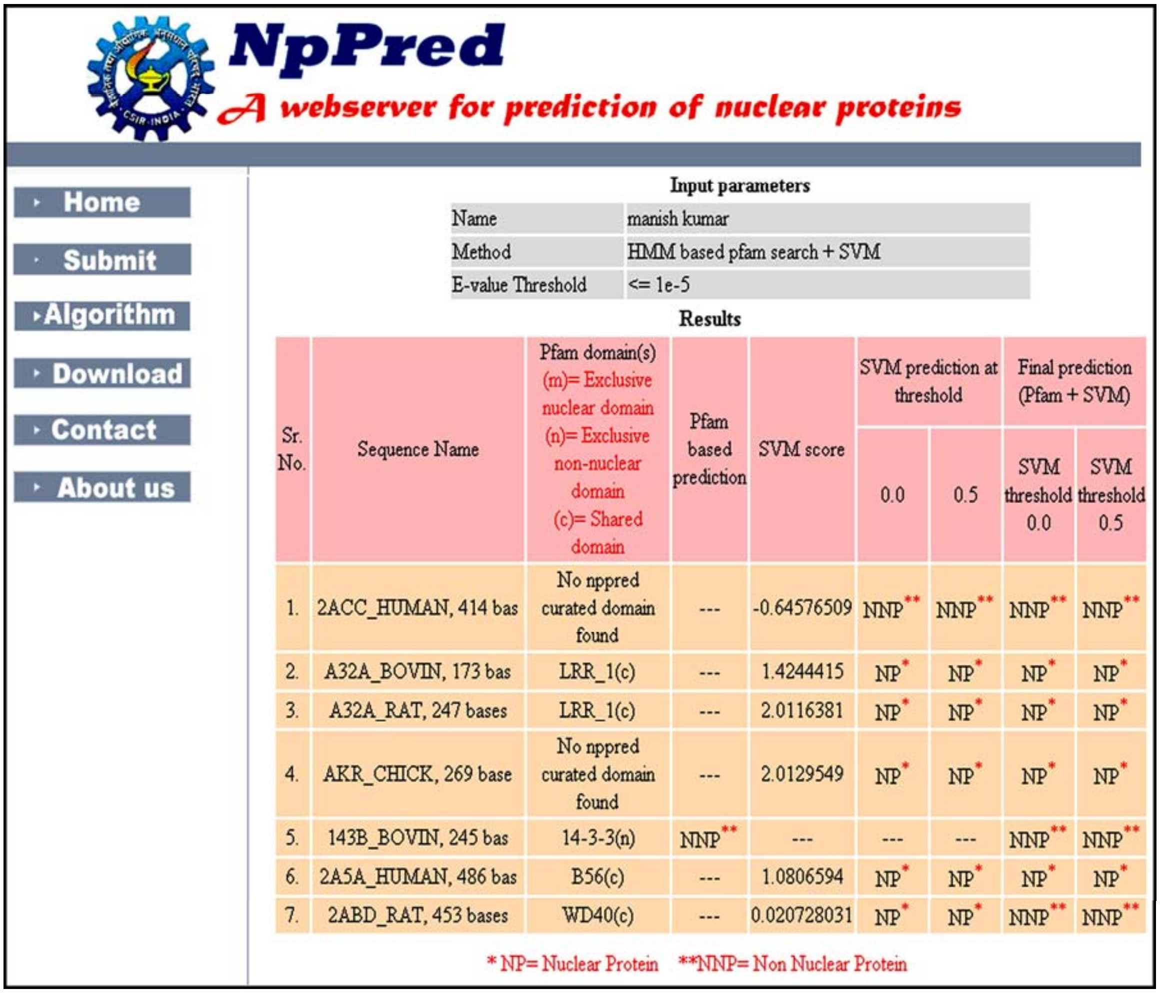
NpPred

A webserver for prediction of nuclear proteins

Home
Submit
Algorithm
Download
Contact
About us

Input parameters

Name	manish kumar
Method	HMM based pfam search + SVM
E-value Threshold	<= 1e-5

Results

Sr. No.	Sequence Name	Pfam domain(s) (m)= Exclusive nuclear domain (n)= Exclusive non-nuclear domain (c)= Shared domain	Pfam based prediction	SVM score	SVM prediction at threshold		Final prediction (Pfam + SVM)	
					0.0	0.5	SVM threshold 0.0	SVM threshold 0.5
1.	2ACC_HUMAN, 414 bas	No nppred curated domain found	---	-0.64576509	NNP**	NNP**	NNP**	NNP**
2.	A32A_BOVIN, 173 bas	LRR_1(c)	---	1.4244415	NP*	NP*	NP*	NP*
3.	A32A_RAT, 247 bases	LRR_1(c)	---	2.0116381	NP*	NP*	NP*	NP*
4.	AKR_CHICK, 269 base	No nppred curated domain found	---	2.0129549	NP*	NP*	NP*	NP*
5.	143B_BOVIN, 245 bas	14-3-3(n)	NNP**	---	---	---	NNP**	NNP**
6.	2A5A_HUMAN, 486 bas	B56(c)	---	1.0806594	NP*	NP*	NP*	NP*
7.	2ABD_RAT, 453 bases	WD40(c)	---	0.020728031	NP*	NP*	NNP**	NNP**

* NP= Nuclear Protein **NNP= Non Nuclear Protein

Figure S4: Prediction result of NpPred with hybrid approach of HMM based Pfam search and SVM.
